# Snare-in-snare technique: An innovative method for leadless pacemaker retrieval

**DOI:** 10.1016/j.hrcr.2025.03.013

**Published:** 2025-03-20

**Authors:** Yuya Nakamura, Yosuke Kai, Rimpei Ueno, Takamasa Ishikawa, Taku Asano, Toshiro Shinke

**Affiliations:** Division of Cardiology, Department of Medicine, Showa University School of Medicine, Tokyo, Japan

**Keywords:** Snare-in-snare technique, Advanced retrieval methods, Leadless pacemakers, Leadless pacemaker retrieval, Device extraction

**Key Teaching Points**

• “Dancing Micra” (excessive device mobility) and abnormal device orientation are major challenges in leadless pacemaker retrieval, sometimes limiting the success of conventional single- or double-snare techniques.
• The snare-in-snare technique enhances retrieval efficiency by combining the EN Snare’s adaptable engagement with the ONE Snare’s precise grasp, enabling stable and controlled device capture.
• The snare-in-snare technique provides a viable alternative for cases in which standard single- or double-snare methods fail, particularly in devices with excessive mobility or suboptimal orientation.
• The snare-in-snare technique has been shown to reduce procedural time, fluoroscopy time, and radiation exposure compared with conventional retrieval methods.

## Introduction

Leadless pacemakers, such as the Micra™ transcatheter pacing system (Medtronic, Minneapolis, MN), have transformed conventional pacemaker therapy by reducing the risks associated with transvenous leads and subcutaneous pockets.^1^^,^^2^ These devices offer a minimally invasive alternative with high implantation success rates and reduced complications.^3^ However, leadless pacemakers still carry certain risks, such as device dislodgement, cardiac perforation, and a low risk of infection. Retrieval and reimplantation are increasingly employed in selected cases. Retrieval techniques, including single- and double-snare systems, have proven effective even for devices implanted for prolonged durations.^4^ However, existing retrieval methods do not always ensure successful retrieval in all cases. Device-related complications such as infection and elevated pacing thresholds, which may necessitate retrieval, highlight the importance of effective retrieval strategies.

The snare-in-snare technique facilitates leadless pacemaker retrieval by combining the adaptable engagement of the EN Snare™ (Merit Medical, South Jordan, UT) with the precise grip of the ONE Snare™ (Merit Medical). This approach enhances procedural control and reduces radiation exposure. This report examines the feasibility and advantages of this technique through 2 illustrative cases.

## Case reports

### Access and preparation of the snare-in-snare technique

The snare-in-snare technique involves the following access and preparation steps:

1. Right femoral vein access: A Micra introducer sheath is used, followed by a 14F sheath and the deployment of the ONE Snare.
2. Left femoral vein access: An Agilis™ NxT (Abbott Cardiovascular, Abbott Park, IL) steerable sheath is used in combination with the EN Snare.

The EN Snare, equipped with 3 loops, allows for adaptable capture of the target object, facilitating an easy initial grasp. However, it is less effective in selectively grasping fine structures of the captured object. In contrast, the ONE Snare features a single loop positioned vertically at its shaft, making it more challenging to position the target within the loop. Nevertheless, once the target object has passed through the loop, the ONE Snare enables selective grasping of specific structures with greater precision. The use of the 14F sheath is recommended to prevent backflow bleeding.

### Description of the snare-in-snare technique

**Figure 1:**
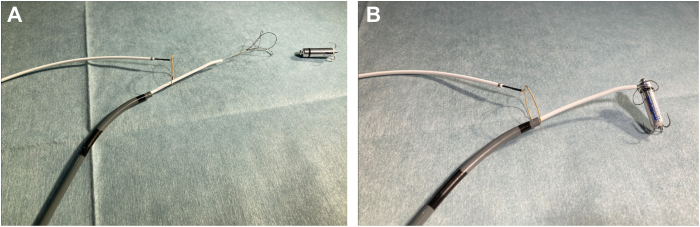
Demonstration of the snare-in-snare technique. **A:** The EN Snare is threaded through the loop of the ONE Snare, preparing to grasp the Micra device. Using the deflection of the Agilis sheath, the EN Snare secures the Micra device with its 3-loop structure, ensuring precise and stable grasping. **B:** After the EN Snare has securely grasped the Micra device, the ONE Snare advances along the EN Snare to grasp the Micra device. In this process, the body of the Micra held by the EN Snare serves as an anchor, allowing the ONE Snare to easily and securely capture the Micra device.

The snare-in-snare technique is first performed by threading the EN Snare through the loop of the ONE Snare within the right atrial space (Figure 1A). The EN Snare, positioned within the Agilis sheath, offers excellent maneuverability, making it easy to securely grasp the body of the Micra device as the initial step. Once the EN Snare stabilizes the device, the ONE Snare is advanced along the EN Snare (Figure 1B). After the loop of the ONE Snare encircles the body of the Micra device, the EN Snare is disengaged from the device. At this point, the ONE Snare, now solely grasping the body of the Micra device, is carefully maneuvered proximally to accurately capture the retrieval head.

This approach facilitates the rapid grasping of the Micra device and ensures precise capture of the retrieval head. The ONE Snare, which securely grasps the retrieval head, pulls the Micra device into the right atrium. The disengaged EN Snare is then used again to grasp the Micra device within the right atrium. By applying countertraction with the EN Snare during sheath insertion and aligning the device coaxially with the sheath, it enables secure and reliable retrieval into the sheath.

## Case 1

**Figure 2:**
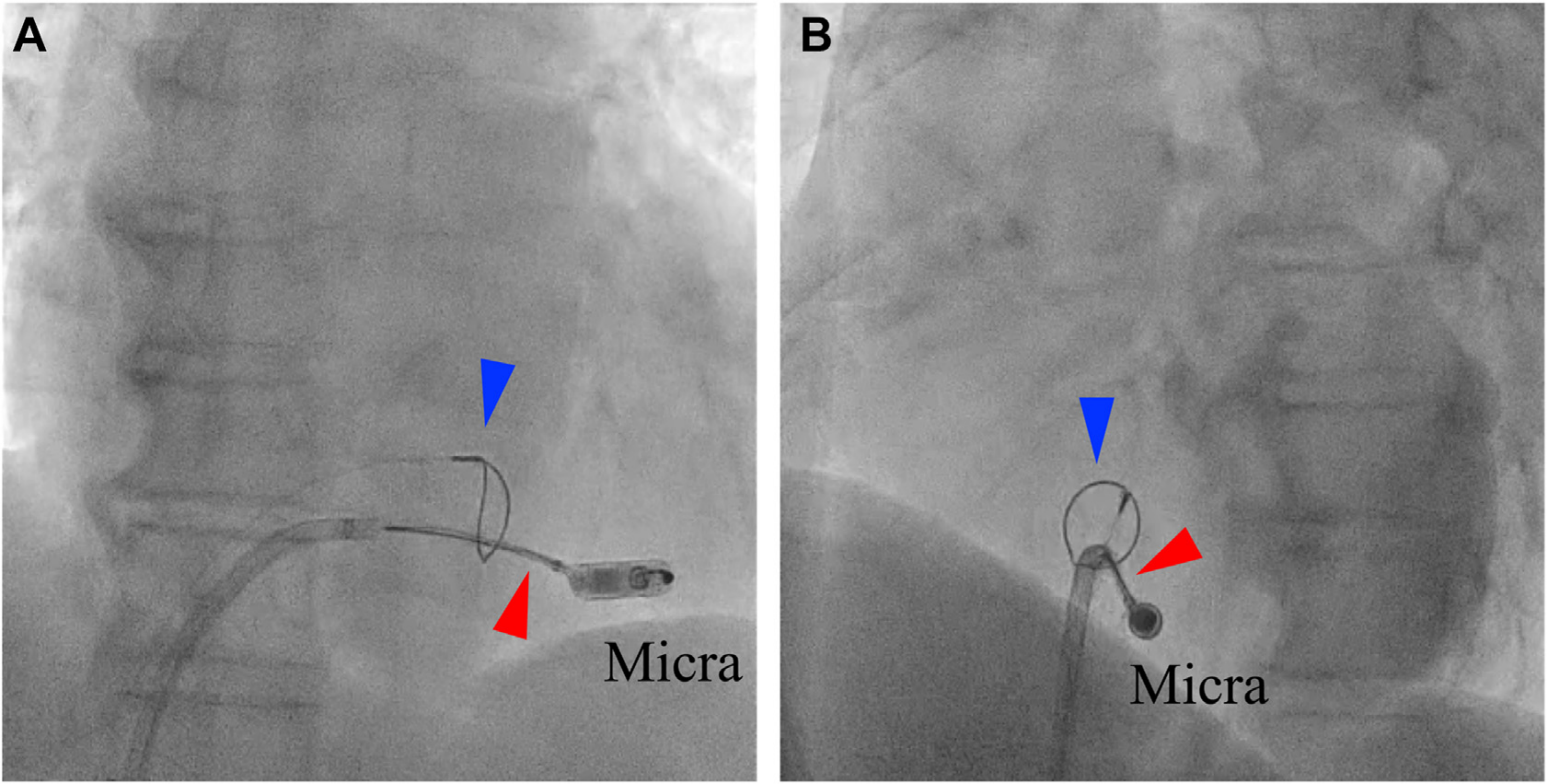
Fluoroscopic images of the snare-in-snare technique in case 1. **A:** AP view and **B:** LAO view showing the EN Snare (*red arrowhead*) already grasping the Micra device after passing through the loop of the ONE Snare (*blue arrowhead*). The ONE Snare is being advanced along the EN Snare to pass around the body of the Micra device. These fluoroscopic images from case 1 demonstrate the coordinated use of both snares during the retrieval process. AP = anteroposterior; LAO = left anterior oblique.

An 81-year-old man with a history of hypertension, dyslipidemia, and Micra transcatheter pacing system implantation for bradycardia-related atrial fibrillation presented with progressive dyspnea on exertion 10 months after the initial Micra implantation. The patient was found to have an increased pacing threshold and pacing failure, prompting the decision for device retrieval and reimplantation. During the procedure, significant mobility of the Micra device was observed, a phenomenon referred to as “Dancing Micra,” which made capturing the retrieval head with a single-loop snare challenging. Additionally, there was concern that the uncertain fixation of the tines could become dislodged during the retrieval attempt, potentially causing the device to migrate into the right ventricular outflow tract. To address this issue, an additional Agilis sheath was introduced via the left femoral vein, and the snare-in-snare technique was employed. The EN Snare, passed through the Agilis sheath, promptly captured the Micra device. The ONE Snare was then advanced along the EN Snare to precisely capture the retrieval head (Figure 2A, 2B). After retrieving the Micra device into the right atrium with the ONE Snare, the EN Snare grasped its distal end to apply countertraction, aligning it coaxially with the sheath for smooth retrieval. The procedure, from anesthesia induction to device removal, was completed in 28 minutes, including 4 minutes of fluoroscopy, with a total radiation dose of 7.1 Gy/cm^2^.

After successful retrieval, a new Micra device was implanted with optimal parameters. The pacing threshold was 0.75 V (0.24 ms), with an R-wave amplitude of 8.5 mV and an impedance of 580 Ω. The patient’s recovery was uneventful, and follow-up showed no recurrence of elevated thresholds.

## Case 2

**Figure 3:**
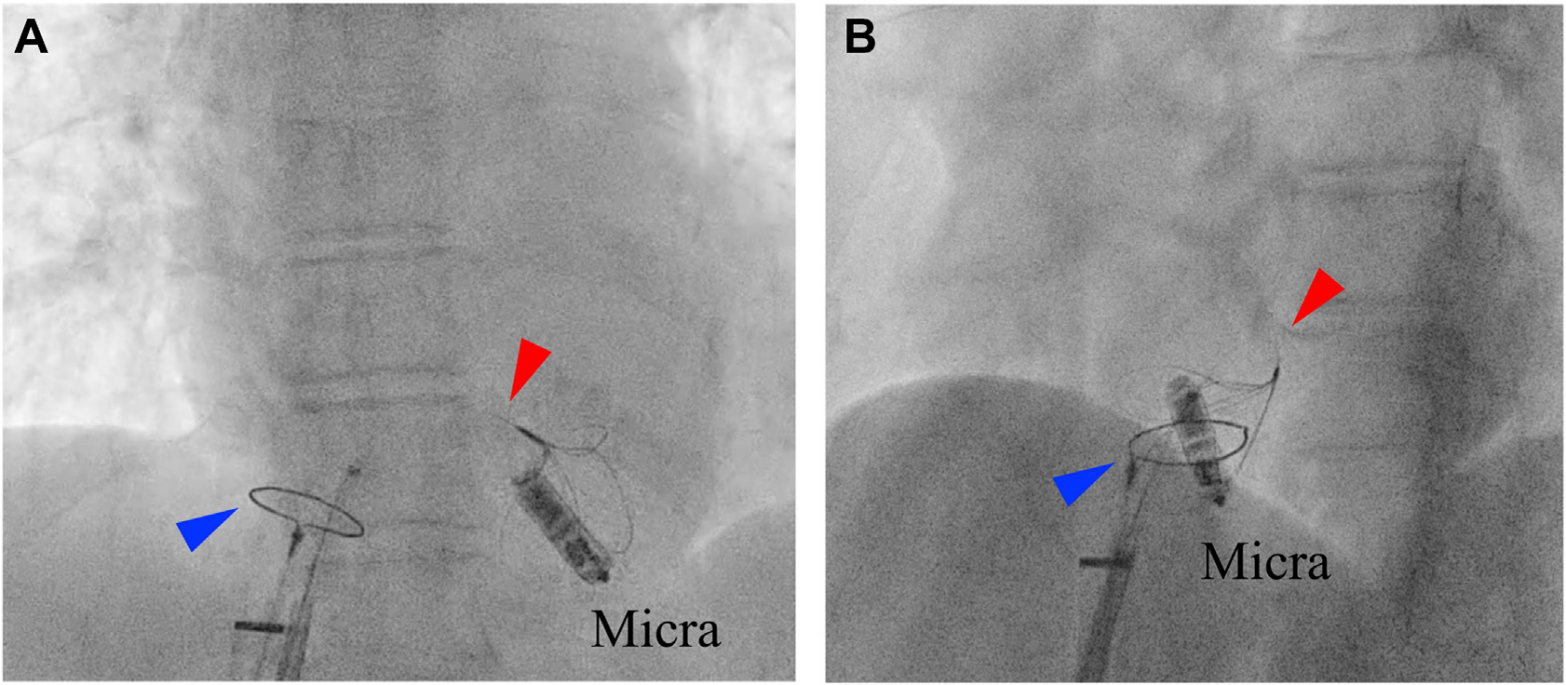
Fluoroscopic images of the snare-in-snare technique in case 2. **A:** AP view and **B:** LAO view showing the EN Snare (*red arrowhead*), threaded through the loop of the ONE Snare (*blue arrowhead*), attempting to capture the Micra device. The Micra device, located near the tricuspid annulus, is being grasped by 1 of the 3 loops of the EN Snare. AP = anteroposterior; LAO = left anterior oblique.

An 86-year-old woman presented to the emergency department with worsening heart failure symptoms 2 months after Micra implantation for advanced atrioventricular block. Electrocardiography showed pacing failure, prompting hospitalization. Imaging demonstrated that the orientation of the body of the Micra device had significantly tilted compared with its original implantation. The retrieval head was located near the base of the right ventricle, wedged below the tricuspid valve. This abnormal tilt led to an increased pacing threshold and pacing failure, making it difficult to capture the retrieval head. Initial attempts using a single-snare technique failed because the tricuspid valve obstructed the passage of the ONE Snare through the Micra device. Consequently, an additional Agilis sheath was introduced, and the snare-in-snare technique was employed for retrieval. The EN Snare effectively captured the Micra device by using the deflection and curvature of the Agilis sheath (Figure 3A, 3B). Using the Micra device secured by the EN Snare as an anchor, the ONE Snare was advanced along the EN Snare to grasp the Micra device. The ONE Snare then successfully pulled the Micra device into the Micra sheath, enabling smooth and safe retrieval. The procedure, from anesthesia induction to device removal, was completed in 25 minutes, including 5 minutes of fluoroscopy, with a total radiation dose of 5.8 Gy/cm^2^. After retrieval, a new Micra device was implanted with optimal parameters, also without complications. The new device achieved a pacing threshold of 0.5 V (0.24 ms), with an R-wave amplitude of 10.5 mV and an impedance of 620 Ω. The patient recovered uneventfully, and follow-up confirmed stable device performance with no recurrence of elevated thresholds.

## Discussion

The snare-in-snare technique represents a notable advancement in the Micra retrieval, particularly in complex cases. In case 1, the technique effectively managed significant device mobility (“Dancing Micra”), whereas in case 2, it addressed difficulties in achieving coaxial alignment because of the device’s unusual angle. By leveraging the EN Snare and Agilis sheath for easier grasping and the precise grip of the ONE Snare, this technique overcomes key limitations of conventional retrieval methods, offering greater efficiency and ease of use. Furthermore, when single- or double-snare techniques prove unsuccessful, the addition of an Agilis sheath via the left femoral vein facilitates the application of the snare-in-snare technique. This adaptability highlights the technique’s versatility and positions it as a critical tool for overcoming complex procedural challenges in leadless pacemaker retrieval.

### Improved efficiency and comparison with existing techniques

The snare-in-snare technique offers substantial improvements in procedural efficiency, reducing procedural time, fluoroscopy time, and radiation exposure compared with conventional methods. For instance, existing single-snare techniques report average procedural times of 89 ± 16 minutes, with fluoroscopy times of 18.0 ± 6.6 minutes.^5^ In our cases, procedural times were reduced to 25 to 28 minutes, and fluoroscopy times were also reduced to 4 to 5 minutes, with radiation exposure ranging from 5.8 to 7.1 Gy/cm^2^. This efficiency minimizes risks for both patients and operators while emphasizing the ease and practicality of the procedure. These improvements are attributed to the synergistic use of the Agilis sheath and EN Snare, which enables rapid grasping of the Micra device, and the ONE Snare, which securely captures the retrieval head with enhanced visibility. By leveraging the complementary strengths of these snares, the technique offers reliable and rapid retrieval even in challenging scenarios. Specifically, the snare-in-snare technique addresses limitations seen with single- and double-snare methods, such as significant device mobility or difficulty achieving coaxial alignment because of the device’s angle.

### Limitations

The snare-in-snare technique, although advantageous, has certain limitations. Advanced encapsulation remains a significant challenge, because dense fibrotic tissue may hinder the ability to secure the device. In such cases, adjunctive tools such as cutting sheaths may be required to facilitate retrieval. Additionally, the use of multiple snares requires the involvement of the assistant operator, increasing the procedural complexity compared with conventional single-snare techniques. The snare-in-snare technique requires precise coordination between the primary and assistant operators, because both snares must be manipulated simultaneously. During the procedure, the assistant operator is needed to secure the EN Snare grasping the Micra device and apply counter-traction, while the primary operator advances the ONE Snare along the EN Snare toward the Micra device. Furthermore, the assistant operator is required to adjust the Micra’s axis using the EN Snare. The snare-in-snare technique requires greater teamwork compared with existing retrieval methods, making it essential to share the procedural steps and conceptual understanding of the technique within the team before the procedure.

## Future directions and clinical implications

Further validation of the snare-in-snare technique in larger and more diverse patient cohorts is required to establish its safety, efficacy, and applicability across various clinical scenarios. As the use of leadless pacemakers continues to expand, the need for reliable retrieval methods becomes increasingly critical. This report highlights the feasibility, safety, and efficiency of the snare-in-snare technique. Its versatility and ability to manage complex cases position it as a promising option for addressing the challenges of difficult Micra retrievals and establishing its role as a reliable alternative in such scenarios.

## Conclusion

The snare-in-snare technique offers a novel, efficient, and reliable method for retrieving leadless pacemakers. By combining the strengths of the EN Snare and ONE Snare, this approach enhances procedural control and reduces procedural time and radiation exposure. Future studies are needed to further validate its application and expand its clinical utility.

## Disclosures

None declared.
